# Cyclophilin C-associated protein (CyCAP) knock-out mice spontaneously develop colonic mucosal hyperplasia and exaggerated tumorigenesis after treatment with carcinogen azoxymethane^1^

**DOI:** 10.1186/1471-2407-9-251

**Published:** 2009-07-24

**Authors:** Emina Emilia Torlakovic, Vicki Keeler, Chang Wang, Hyun J Lim, Leslie Ann Lining, Suzanne Laferté

**Affiliations:** 1 Department of Pathology, Royal University Hospital, College of Medicine, University of Saskatchewan, Saskatoon, Canada; 2 Department of Community Health & Epidemiology, University of Saskatchewan, Saskatoon, Canada; 3 Department of Biochemistry, University of Saskatchewan, Saskatoon, Canada

## Abstract

**Background:**

The discovery of a "serrated neoplasia pathway" has highlighted the role of hyperplastic lesions of the colon as the significant precursor of colorectal adenocarcinoma. In mice, hyperplasia of the colonic mucosa is a regular phenomenon after a challenge with colonic carcinogens indicating that mucosal hyperproliferation and thickening, even without cytological dysplasia, represents an early pre-malignant change. Cyclophilin C-associated protein (CyCAP) has been described to down-modulate endotoxin signaling in colorectal murine mucosa and is a murine orthologue of the tumor-associated antigen 90 K (TAA90K)/mac-2-binding protein.

**Methods:**

Female Balb/c wild-type (WT) and CyCAP knock-out (KO) mice (6–8 weeks old) were administered 2 or 6 weekly subcutaneous injections of azoxymethane. The animals were evaluated post-injection at six weeks for aberrant crypt foci (ACF) study and at five months for colon tumor measurement. The thickness of the colon crypts was measured in microns and the number of colonocytes per crypt was also determined in well-oriented crypts. Morphometric analyses of the colon mucosa were also performed in untreated 6–8 weeks old KO and WT animals. Formalin-fixed/paraffin-embedded colon sections were also studied by immunohistochemistry to determine the Ki-67 proliferation fraction of the colon mucosa, β-catenin cellular localization, cyclin D1, c-myc, and lysozyme in Paneth cells.

**Results:**

Cyclophilin C-associated protein (CyCAP)^-/- ^mice, spontaneously developed colonic mucosal hyperplasia early in life compared to wild-type mice (WT) (p < 0.0001, T-test) and crypts of colonic mucosa of the (CyCAP)^-/- ^mice show higher proliferation rate (p = 0.039, Mann-Whitney Test) and larger number of cyclin D1-positive cells (p < 0.0001, Mann-Whitney Test). Proliferation fraction and cyclin D1 expression showed positive linear association (p = 0.019, Linear-by-Linear Association). The hyperplasia was even more pronounced in CyCAP^-/- ^mice than in WT after challenge with azoxymethane (p = 0.005, T-test). The length of the crypts (r = 0.723, p = 0.018, Spearman Correlation) and the number of colonocytes per crypt (r = 0.863, p = 0.001, Spearman Correlation) in non-tumorous areas were positively associated with azoxymethane-induced number of tumors. CyCAP^-/- ^developed larger numbers of tumors than WT animals (p = 0.003, T-Test) as well as overall larger tumor mass (p = 0.016, T-Test). Membranous β-catenin was focally overexpressed in KO mice including proliferative zone of the crypts.

**Conclusion:**

CyCAP^-/- ^represent the first described model of spontaneous colonic mucosal hyperplasia. We conclude that CyCAP-deficient mice spontaneously and after challenge with carcinogen develop significantly more colorectal mucosal hyperplasia, an early stage in murine colonic carcinogenesis.

## Background

Colorectal cancer remains a significant cause of morbidity in Western countries [1]. Colorectal carcinoma evolves through multiple pathways (recently reviewed by Jass J. R.)[2]. For many years clinical practice has been focused on early detection and removal of adenomatous polyps by screening colonoscopy is an important strategy for reducing the risk of developing colon cancer [3]. Colorectal adenoma is considered to be a precursor of about 70% colorectal carcinomas, those that are characterized by chromosomal instability and are microsatellite stable. It appears that as many as 20% of colorectal carcinomas may originate by serrated neoplasia pathway [2]. It has become apparent that clinical management of lesions traditionally diagnosed as "hyperplastic polyps" and generally considered as benign lesions with no malignant potential, may need to change because of the malignant potential associated with some types of serrated polyps including so-called "traditional serrated adenoma" and "sessile serrated adenoma" [1,4-6].

Hyperplasia of the human colon is found in several forms including focal mucosal hyperplasia in hyperplastic polyp, the thickening of the mucosa surrounding colon carcinoma or adenoma, which is termed "transitional mucosa", and regenerative hyperplasia, which may occur in a variety of inflammatory conditions. All of these lesions are considered to be benign, harmless lesions, with no proven premalignant potential [7-9]. Sessile serrated polyp is a newly described type of serrated polyp, now recognized as a main actor in a so-called "serrated pathway" of colon carcinogenesis and it shares many features with the common hyperplastic polyp [5,10,11]. Therefore in humans, two types of colonic serrated mucosal hyperplasia can be distinguished; non-preneoplastic and preneoplastic. Most forms of mucosal thickening of the colon are due to reactive epithelial hyperplasia and do not lead to malignancy, which is in contrast to mice in which mucosal hyperplasia develops as the first change and also in parallel with colonic epithelial tumors after the treatment with carcinogens [12].

The above observations in humans are in agreement with the notion that not all hyperplastic agents have tumor-promoting activity [13]. For instance, TGF- alpha is a potent *in vivo *epithelial mitogen which promotes dramatic hyperplasia in the colon, but does not lead to any alteration in colonic tissue architecture or colonic tumors [14]. TGF- alpha was also found to be absent in ACF in rats and there was reduced amounts of TGF- alpha expression in the upper part of the aberrant crypts compared with the surrounding normal crypts [15]. Although the absence of TGF-alpha was unexpected, it was in accordance with observations from recent *in vitro *studies which demonstrated that the presence of TGF-alpha in the top of the colonic crypt serves to inhibit growth of differentiated colonic epithelial cells [16,17]. In humans, lower levels of TGF-alpha are present in the distal part of the colon, which is the most common site of tumor formation [18]. Additionally, non-preneoplastic secondary mucosal hyperplasia as a reaction to a colonic tumor, so-called "transitional mucosa", possibly develops due to tumoral secretion of IL-15 [19].

Despite these findings, it has been established that thickened and serrated colonic mucosa at least in the form of sessile serrated adenoma (synonymously referred to as "atypical hyperplasia"), represents a morphologic entity of the serrated neoplasia pathway [2,20]. The discovery of different subtypes of serrated polyps enabled morphologic separation of the harmless hyperplastic lesions from those that are likely to be precancerous [4,6]. It is possible that precancerous serrated polyps of the colon represent a human homologue of the murine pre-neoplastic hyperplasia induced by carcinogens since these represent the only form of "hyperplasia" of human colonic mucosa that has been biologically linked to colorectal carcinoma [2].

The tumor-associated antigen 90 K (TAA90K) [21,22], also known as Mac-2-binding protein or 90 K, is a secreted 90–100 kDa glycoprotein widely expressed in human fluids [23], tumor derived cell lines including those with epithelial differentiation [24-28] and elevated in the serum of cancer patients [Iacobelli *et al*., 1993; Natoli *et al*., 1993; Iacobelli *et al*., 1994; Greco *et al*., 2004] as well as sufferers from other nonmalignant diseases such as AIDS [29,30]. It is a member of the scavenger-receptor cysteine-rich domain superfamily implicated in development and regulation of the immune system [31]. On the other hand, its aberrant expression by epithelial neoplastic cells has also been described. Elevated expression of TAA90K has been detected in breast, gastric, lung and pancreatic cancers [32-35] and, in the cases of lung cancer and malignant mesothelioma [33,34], protein levels correlated with metastasis and predicted survival or disease manifestation. In a recent study [36], we have also detected elevated TAA90K expression in colorectal adenocarcinoma. Although the function of aberrantly expressed TAA90K in cancer is unclear, it has been shown to mediate β_1_-integrin-dependent cell adhesion [37], interact with various extracellular proteins including extracellular matrix proteins and galectins [36-38], and stimulate expression of promatrilysin [39], previously implicated in colon cancer progression [40].

However, the primary and physiological role of TAA90K appears to be, according to the current knowledge, in immune regulation. This glycoprotein has been shown to be a potent immune stimulator with positive effects on the generation of cytotoxic effector cells (NK/LAK) from human peripheral blood mononuclear cells (PBMC) [41,42]. In fact, 90 K in human milk may be protective against acute respiratory infections [43]. The protective effect seems to be related to the amount of 90 K ingested during the first few days, as milk 90 K concentrations subsequently decline rapidly in all mothers [44]. These findings suggested that 90 K initiates a defense mechanism in the infant early in life.

In recent years, cyclophilin C-associated protein (CyCAP) has been identified as the murine orthologue of TAA90K with up to 80% homology in their amino acid sequence [45,46]. CyCAP is a widely expressed secreted glycoprotein that modulates the host response to bacterial endotoxin [47]. In response to endotoxin, CyCAP-deficient mice overproduced interleukin 12 and interferon-γ systemically and tumor necrosis factor α locally, proinflammatory molecules that also promote T helper 1 responses. Furthermore, macrophages stimulated *in vitro *with endotoxin in serum deficient CyCAP-null cells secreted more TNF-α, supporting the proposal that CyCAP specifically down-modulates endotoxin signaling [47].

In this report, we have examined the contribution of CyCAP to colon hyperplasia and tumorigenesis *in vivo *before and after injection of the colon carcinogen azoxymethane as a possible animal model suitable for elucidating the role of TAA90K in colon tumorigenesis and progression, thereby providing a level of biological understanding not readily available in clinical disease.

## Methods

### Animals and azoxymethane treatment

Female Balb/C mice (6–8 weeks) were obtained from the Animal Resources Center (University of Saskatchewan, Saskatoon, SK). Breeding pairs of Balb/C CyCAP knock-out mice were obtained from Dr. I. Weissman (Stanford University, San Francisco, CA) and used to set up an in-house breeding colony. Forty seven animals were tested routinely for the presence of pathogens. In particular, mice were free of *Citrobacter freundii*, a bacterium associated with transmissible intestinal hyperplasia [48]. All experimental procedures were carried in accordance with guidelines from the Canadian Council on Animal Care.

Forty seven female Balb/c wild-type and CyCAP knock-out mice (6–8 weeks old) were administered 6 weekly subcutaneous injections of azoxymethane (10 mg/kg) (Midwest Research Institute, MO) [49] for measurements of ACF or colon tumor incidence, respectively, using procedures described previously [50]. Six weeks (ACF measurement) or five months (colon tumor measurement) post-injection, animals were sacrificed, the intestine removed and washed in ice-cold PBS. Colons were cut longitudinally, laid flat between two pieces of 3 mm filter paper and fixed for at least two hours in formalin (10% formaldehyde in phosphate buffered saline pH 7.0). For ACF and tumor measurements, colons were stained for 5 min in a solution of 1% methylene blue in PBS and destained for 5 min in PBS. Stained colons were examined microscopically for the presence of ACF, as described by Bird *et al*. [51] and also for the presence of tumors. The size of tumors (length in mm × width in mm) was measured at a magnification of 40× using a gradicule (Precision Instruments, Vancouver, BC) calibrated with a slide micrometer. After the data were collected, the fixed colons of 5 untreated and 5 azoxymethane-treated wild-type and CyCAP knock-out animals were embedded in paraffin. Hematoxylin and eosin-stained five micron-thick serial sections were examined histologically.

### Measurements and cell counts

All measurements and cell counts were performed on histologically non-tumorous mucosa, with no definite pathological findings. The exact absolute length of each colonic crypt was measured in μm and the exact absolute number of cells per each crypt was recorded. In addition, only areas away from the tumors and lymphoid aggregates were measured to avoid so-called traditional mucosa or reactive changes. The thickness of the crypts was measured in microns in 60 to 246 well-oriented crypts (all available well-oriented crypts were measured) using a calibrated camera (Nikon Digital Sight DS-SM, Japan) mounted on the microscope (Nikon Eclipse 80i, Japan). The presence of the full thickness of the crypt with open lumen, to avoid tangentionally-sectioned crypts, was the only positive selection criterion. The same method was used for establishing the crypt proliferation fraction as measured by the number of Ki-67+ cells divided by the total number of cells per crypt. Immunohistochemical tests for Ki-67, cyclin D1, c-myc, lysozyme, and β-catenin were done as previously described [52,53]. In the majority of animals, a total of 60 accounted for most of the well-oriented crypts. The number of colonocytes per crypt was also determined in at least 60 well-oriented crypts per animal. B-catenin was evaluated for cellular localization and its nuclear, cytoplasmic, and membranous was recorded. The intensity of staining and its distribution was recorded in 60 well-oriented crypts using semiquantitative scale (0-negative, 1-weak membranous staining, and 2-strong membranous staining), as well as the anatomical location of the positive cells in the mucosa (crypt base, crypt body, and colonic plate).

### Statistical analysis

Descriptive statistics were used to summarize data. Initial descriptive analyses were done to determine whether to use parametric t-test or non-parametric Mann-Whitney test for mean comparisons and whether to assume equal variance or unequal variance. A Student's t-test was used to examine the differences for continuous variable under assuming either equal or unequal variance. Association between variables were determined using Spearman correlation coefficient analysis. Linear regression model were also used after checking assumption of linearity, normality and constant variance of independent error. A p-value of less than 0.05 was considered statistically significant. The analysis was performed using the SPSS – 15 software.

## Results

Correlation between the number of cells per crypt and the length of the crypt was significant (p < 0.0001, Spearman Correlation). The coefficient of correlation was 0.86. This result indicated that the increased length of the crypts was due to hyperplasia, rather than hypertrophy or other phenomena like edema or various artifacts.

**Figure 1:**
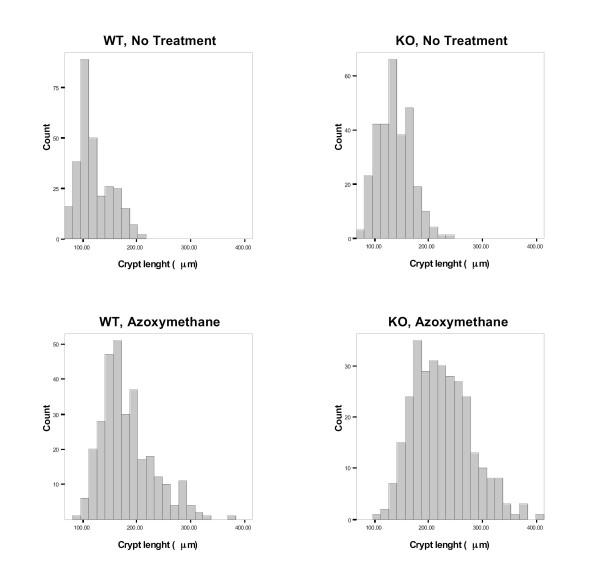
**Distribution of colonic mucosal measurements in WT and KO mice**. Histograms of measurements illustrate distribution of crypt length in wild type and CyCAP^-/- ^animals.

**Figure 2:**
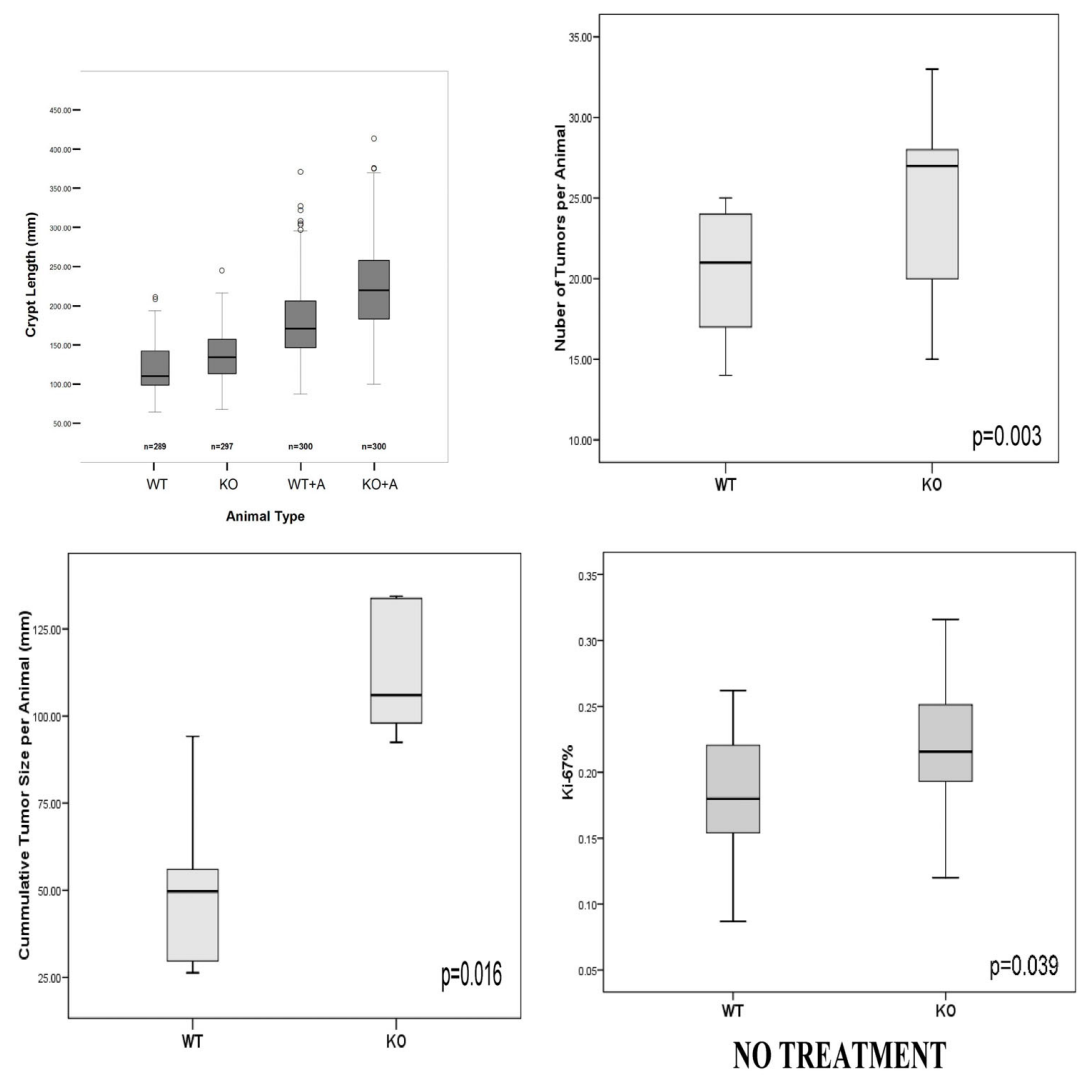
**The differences in the measured crypt length were significant before and after treatment by azoxymethane**. CyCAP^-/- ^animals spontaneously developed increased crypt length and also developed much more exuberant response to azoxymethane treatment (**A**), which resulted in more hyperplasia (**A**) larger number of adenomas (p = 0.003, Linear Regression) (**B**) and increased cumulative size of adenomas (p = 0.016, Linear Regression) (**C**). The CyCAP^-/- ^untreated animals also spontaneously developed higher crypt proliferation ratio (p = 0.039, Mann-Whitney Test) (**D**).

**Figure 3:**
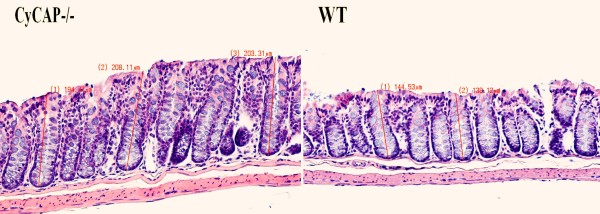
**The CyCAP^-/- ^mice spontaneously develop colonic mucosal hyperplasia early in life at 6–8 wk**. The difference between this hyperplastic mucosa and normal mucosa of the WT mice is inconspicuous by superficial observation, but statistically, the difference is significant (p < 0.0001, T-test) (20 × magnification, H&E).

**Figure 4:**
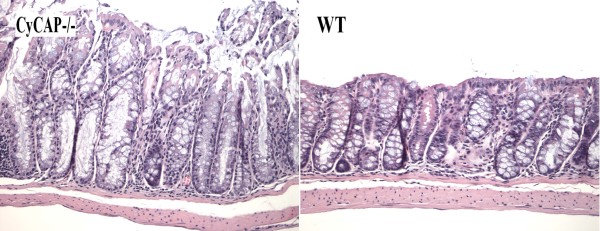
**The differences in the crypt length and crypt cell content are more obvious between WT and KO animals after the treatment by azoxymethane**. After the treatment by azoxymethane, the CyCAP-/- mice develop more extensive mucosal hyperplasia of the colon than WT animals (p < 0.0001, T-test). The mononuclear inflammatory content in the lamina propria is not increased compared to WT animals (20 × magnification, H&E).

**Figure 5:**
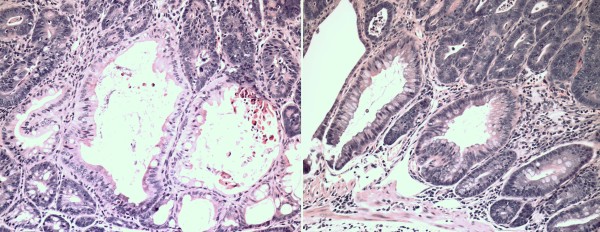
**In many early adenomas after azoxymethane treatment, hyperplastic-like changes were also present**. Adenomatous changes without (**A**) and with (**B**) evidence of cytologic dysplasia and cystically dilated crypts and hyperplastic changes in close proximity to muscularis mucosae were found in tumors after azoxymethane treatment (20 × magnification, H&E).

**Figure 6:**
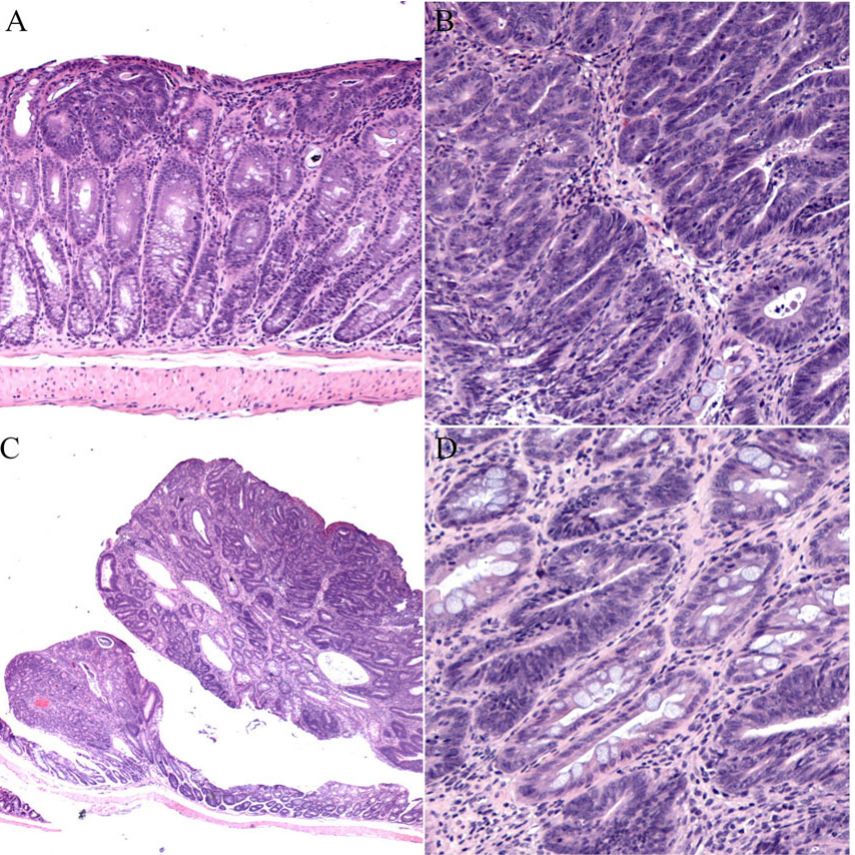
**Spectrum of histologic changes in KO mice treated by azoxymethane**. Early dysplastic changes were noted in many areas, some of which corresponded to ACF (A). More extensive moderate dysplasia was noted in areas of thickened mucosa (B) and in tumors/adenomas (C). Focal severe dysplasia/carcinoma in situ changes were also note (D).

All available well-oriented crypts were evaluated for the number of cells per crypt as well as for the absolute length of the crypt. The number of observations (N) per animal varied from 60 to 246 per animal depending on the number of available well-oriented crypts. The results are summarized in Tables 1 and 2 and Figure 1, 2, 3, 4, 5, 6, and 7. The length of the crypts in CyCAP null mice and WT mice (r = 0.723, p = 0.018, Spearman Correlation) as well as the number of colonocytes per crypt (r = 0.863, p = 0.001, Spearman Correlation) in non-tumorous areas of the colon were positively associated with total number of tumors induced by azoxymethane (Figure 1 and 2). Also, the length of the crypts (r = 0.782, p = 0.008, Spearman Correlation) and the number of colonocytes per crypt (r = 0.927, p < 0.0001, Spearman Correlation) in non-tumorous areas of the colon of CyCAP null mice and WT mice correlated with the total size of the tumors. In addition, untreated 8-week old CyCAP null mice had significantly thicker mucosa and significantly larger numbers of colonocytes per crypt (hyperplasia) (p < 0.0001, T-test), as well as higher proliferation fraction as measured by Ki-67 immunohistochemical test (p = 0.032, Mann-Whitney Test) even before azoxymethane treatment was initiated (Figure 1, 2A, and 3) indicating spontaneous development of mucosal hyperplasia. The difference in diffuse mucosal thickness was even more pronounced after the treatment with carcinogen (p < 0.0001, T-test, Figure 2A, 3, 4, and 5). Similarly, the null mice had larger numbers of cells per crypt both before (p = 0.032, Linear Regression; plots not shown) and after treatment with carcinogen (p = 0.012, Linear Regression; plots not shown) compared with WT animals.

**Figure 7:**
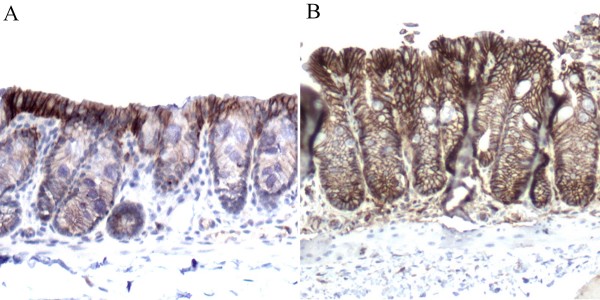
**B-catenin cellular localization in WT and CyCAP KO mice**. In WT mice, β-catenin expression is higher in the colonic plate than in the crypts of the colon mucosa (A). However, CyCAP KO mice show overall increased membranous expression of β-catenin with irregular distribution, which focally significantly extended into crypts (B). No definite nuclear localization of β-catenin was detected either in WT or KO mice.

**Table 1:** Distribution of Mean Thickness and SD of Non-Tumoral Colonic Mucosa

Animal Type	Mean (μm)	SD
**Type 1: **WT + A	182.5761	48.53183
**Type 2: **KO + A	225.4123	54.00269
**Type 3: **WT	121.3074	30.88762
**Type 4: **KO	136.7142	30.33236

SD = Standard Deviation, A = Azoxymethane, WT = Wild Type Mice, KO = Knock Out Mice

**Table 2:** Independent Samples T-Test for Equality of Means

Animal Type*	Sig. (2-tailed)	Mean Difference	Std. Error Difference	Lower 95% CI**	Upper 95% CI**
**Type 1 vs. 2**	**< 0.0001**	42.83620	4.19191	34.60355	51.06885
**Type 3 vs. 4**	**< 0.0001**	15.40680	2.52900	10.43976	20.37384
**Type 1 vs. 3**	**< 0.0001**	61.26872	3.36607	54.65772	67.87973
**Type 2 vs. 4**	**< 0.0001**	88.69812	3.58972	81.64806	95.74819

* Animal type as per Table 1. ** Confidence Interval of the Difference

CyCAP null mice had significantly larger numbers of ACF and tumors per animal than control WT animals (p = 0.016, T-Test, Figure 2B). The mean tumor size was larger in CyCAP null mice (p = 0.031, T-Test) as well as cumulative tumor size per animal (p = 0.003, T-Test, Figure 2C). Spectrum of histologic changes is shown in Figure 6.

Beta-catenin was not detected in the nuclei of the colonocytes either in WT or KO mice. Membranous β-catenin was overall less expressed in WT mice than in KO mice (p < 0.0001, Mann-Whitney Test). In WT mice, β-catenin was very weakly expressed in the colonic crypts with more notable expression in the colonic plate/luminal surface of the mucosa (Figure 7A). Membranous β-catenin in CyCAP KO mice is not only overall stronger, but also there is strong expression not only in the colonic plate, but focally also in an irregular fashion including the base of the crypts (Figure 7B). The difference between WT and KO mice in the distribution of β-catenin expression was also highly significant (p < 0.0001, Mann-Whitney Test). This overexpression of the membranous β-catenin is highly variable from one crypt to another. No definite difference in c-myc expression between WT and KO mice was noted. Cyclin D1 expression was significantly higher in KO mice (p < 0.0001, Mann-Whitney Test) and also positively correlated with Ki-67 expression (p = 0.019, Linear-by-Linear Association).

## Discussion

Historically, it has been claimed that the transition from hyperplasia to neoplasia is frequent [54,55]. Human studies and experimental data from animals suggest that high rates of colonic epithelial cell replication enhance the development of colon cancer [56-58]. Exaggerated mucosal proliferative activity is in some way associated with the development of large-bowel neoplasia. Individuals with inflammatory and proliferative diseases of the bowel such as Crohn's disease, ulcerative colitis, and familial polyposis suffer an unusually high risk and early onset of colorectal cancer. However, as mentioned above, this has never been shown in a very common form of colonic mucosal hyperplasia, i.e. hyperplastic polyps [59] and no evidence of diffuse hyperplasia was ever detected in patients with colorectal carcinoma despite demonstrable abnormalities in cell kinetics in histologically normal mucosa in some patients with adenocarcinoma [60] adenomatous polyps [61], familial polyposis [62], and ulcerative colitis [63].

The hyperplasia-neoplasia model has also been challenged at least by one experiment in rodents [64] in which experimentally produced megacolon was associated with reactive mucosal hyperplasia, but showed less malignant tumors than rats without megacolon, both treated by dimethylhydrazine for 20 weeks. One possible interpretation of these results would suggest that experimentally induced reactive hyperplasia is not a premalignant condition and may even be protective of neoplastic transformation. This is in contrast with our findings of increased number of tumors developing in animals with more pronounced mucosal hyperplasia suggesting that colorectal hyperplasia in our model is a preneoplastic change.

In mice, colonic mucosal hyperplasia can be caused by infection. Transmissible murine colonic hyperplasia is the only naturally occurring hyperplastic disease of the colon in mice [65]. This severe but transient mucosal hyperplasia of the distal colon is caused by *Citrobacter freundii *[48] and was shown to have an expansion of the proliferative zone, an increased labeling index, a prolongation of the cell cycle time, an increase in variation of DNA synthesis times resulting in prolongation of the S phase, and an accelerated migration rate [66]. Interestingly, these findings parallel the "atypical" kinetics found in human and murine preneoplastic and neoplastic disorders of the colon, and also abnormal proliferative kinetics which occur in the flat mucosa of humans between existing tumors or polyps and in the preneoplastic mucosa of rodents treated with carcinogens [60,62]. Hyperplasia, analogous to tumor promoters which induce proliferation [12], has been shown to reduce the lag period for the appearance of carcinogen-induced neoplastic lesions and to allow their induction with single, subthreshold doses of carcinogen. Our data indicate that the CyCAP null mice spontaneously develop thicker and more proliferative mucosa of the colon, which allowed for marked induction of both additional mucosal hyperplasia and mucosal tumors after the treatment with carcinogen and is in agreement with previous studies, which have indicated that mucosal hyperplasia has a profound effect upon early carcinogenesis [67,68].

Intraluminal factors including western-style diet/food were found to produce colonic mucosal hyperplasia in experimental animals [69,70]. However, Delvaux et al. [71] detected an increase in colonic cell proliferation within an excluded colostomy segment. It was also recently demonstrated that serum insulin levels directly correlate with the presence of adenoma and hyperplastic polyps in the proximal colon of humans [72]. The history of tobacco smoking was also associated with higher incidence of hyperplastic polyps [59]. The latter studies suggested that extraluminal factors may also play an important role in the regulation of colonic mucosal growth. Immune regulation and host defense may represent one additional extraluminal factor in regulation of colonic mucosal growth. Suzuki *et al*. [73] developed an experimental hyperplasia in nu/nu mice by transfer of lymphocytes from the C57BL/6 mice infected by murine leukemia virus (LP-BM5). Lymphocyte transfer to nu/nu mice induced colitis and hyperplasia of intestinal epithelial cells. In contrast to CyCAP null mice in our study, which did not show any mucosal inflammation in addition to the hyperplasia, these mice developed marked mononuclear infiltrates rich in CD4+/CD45RB^low ^T-cells. Suzuki *et al*. [73] work shows that in such model, hyperplasia of the colon may be due to defects in immunoregulatory mechanisms. TAA90K belongs to an ancient protein superfamily which is defined by a scavenger receptor cysteine-rich (SRCR) domain [41]. It is particularly noteworthy that all the previously characterized SRCR domains occur in proteins involved in or thought to participate in critical functions of the cellular host defense system, and thus they resemble the immunoglobulin superfamily of the cysteine-containing protein domains (reviewed in ref. 31). Histochemically, Mac-2 has been located intracellularly in many members of the macrophage lineage, including tissue macrophages, certain dendritic cells, alveolar and Kupffer cells [74,75]. Importantly, Jallal et al. [23] showed that engineered enhancement of 90 K expression results in significant (> 80%) tumor growth inhibition, not by direct action on the tumor cells, but by stimulation of the residual cell-mediated immune defense of the nude mouse.

Recently, Rigby at al. [76] showed a role of suppressor of cytokine signaling-3 (SOCS3) in colonic mucosal epithelial proliferation. Mice with disrupted SOCS3 gene responded with increased proliferation and increased crypt depth after acute dextran sodium sulfate(DSS)-induced mucosal injury or after azoxymethane and chronic DSS. However, the authors did not report spontaneously developing mucosal hyperproliferation and mucosal hyperplasia as it was the case in CyCAP deficient mice in our study. Similarly, Jones et al. [77] reported that conditional deletion of beta-1 integrins in the intestinal epithelium causes a loss of Hedgehog expression and intestinal hyperplasia. However, images illustrated in their publication reveal architectural abnormalities and cytologic atypia, both of which are indicating development of ectopic crypts and adenomas rather than simple lengthening of the crypts, which would indicate hyperplasia only.

Wnt/β-catenin signalling is critically important for colon organogenesis and also for colon carcinogenesis. Wnt protein binding to Frizzled (Fzd) and low-density lipoprotein receptor-related proteins (LRP) causes inactivation of glycogen synthase kinase (GSK) 3β and Axin, respectively. The inactivation of these proteins stabilizes β-catenin, which subsequently accumulates in the cell nucleus and activates the transduction of target genes that are crucial in the G1-S-phase transition and therefore, nuclear β-catenin co-localize with markers of proliferation including c-myc and cyclin D1, and Ki-67 [78,79]. This pathway also has a critical role in Paneth cell differentiation [80]. We have not observed any morphological or immunohistochemical evidence of increase or aberrant localization of Paneth cells in the colon specimens of the CyCAP knockout mice. Also, no abnormalities of Paneth cells were identified in the small intestine of the CyCAP KO mice. While β-catenin was not detected in the nuclei of the colonocytes in either WT or KO mice and there were no definite difference in expression of the c-myc and cyclin D1, KO mice had increased membranous β-catenin expression compared to their matched WT control and strong expression of β-catenin was also detected in all segments of the colonic crypts including the base indicating that proliferative zone shows β-catenin phenotype that is seen only on the colonic plate of normal mice. Since one way by which cytoplasmic β-catenin is prevented from returning to the nucleus is by being locked by the emerging E-cadherin into adherens junctions which link the cell to proliferatively shut-down functioning cells moving up and out of the crypt, the finding of overexpressed membranous β-catenin in proliferative compartment of the hyperplastic mucosa may represent a significant abnormality; however, it needs to be further evaluated in future studies.

## Conclusion

CyCAP knockout mice develop mucosal hyperproliferation and mucosal hyperplasia early in life without exposure to carcinogen and as such they represent the first known model of spontaneous colonic hyperplasia. When challenged by a carcinogen, CyCAP knockout mice also develop significantly more hyperplasia and more tumors than wild-type mice, indicating that their mucosa is more proliferative than in WT animals. Therefore, our results link an early premalignant change in murine colorectal mucosa to deficiency of a glycoprotein with a role in modulation of endotoxin signalling. Further studies are necessary to elucidate if this multifunctional glycoprotein may also have a role in human preneoplastic hyperplasia, the pathogenesis of which is largely unknown.

## Competing interests

The authors declare that they have no competing interests.

## Authors' contributions

EET participated in the design of the study, carried out morphometric and immunoassays, participated in statistical analysis, and drafted the manuscript. VK helped with animal experiments. CW participated in the histological analyses. HJL participated in the design of the study and the statistical analysis. LAL evaluated and quantitated Ki-67 proliferation fraction. SL conceived of the study, performed experiments with laboratory animals, participated in its design and coordination and helped to draft the manuscript. All authors read and approved the final manuscript.

## Pre-publication history

The pre-publication history for this paper can be accessed here:

http://www.biomedcentral.com/1471-2407/9/251/prepub
